# A Multi-Feature Fusion Method for Life Prediction of Automotive Proton Exchange Membrane Fuel Cell Based on TCN-GRU

**DOI:** 10.3390/ma17194713

**Published:** 2024-09-25

**Authors:** Jiaming Zhang, Fuwu Yan, Changqing Du, Yiming Zhang, Chao Zheng, Jinhai Wang, Ben Chen

**Affiliations:** 1 Hubei Key Laboratory of Advanced Technology for Automotive Components, Wuhan University of Technology, Wuhan 430070, China; 240408@whut.edu.cn (J.Z.);; 2 Hubei Research Center for New Energy & Intelligent Connected Vehicle, Wuhan University of Technology, Wuhan 430070, China; 3 National Energy Key Laboratory for New Hydrogen-Ammonia Energy Technologies, Foshan Xianhu Laboratory, Foshan 528200, China

**Keywords:** PEMFC, life prediction, multi-feature fusion method, TCN-GRU

## Abstract

The Proton Exchange Membrane Fuel Cell (PEMFC) is a fast-developing battery technology, and the key to its reliability and lifespan improvement lies in the accurate assessment of durability. However, the degradation mechanism of the PEMFC is hard to determine and its internal parameters are highly coupled. Thus, the development of a more accurate life prediction model that meets the actual scenarios is to be investigated urgently. To solve this problem, a multi-feature fusion life prediction method based on the Temporal Convolutional Network-Gated Recurrent Unit (TCN-GRU) is proposed. A TCN algorithm is used as the prediction base model, and two GRU modules are included with the model to strengthen the model’s expression ability and improve its predictive accuracy. Two widely recognized datasets and two operating conditions are utilized for model training and prediction, respectively. Comparisons are made with single-feature parameter models in terms of Root Mean Square Error (RMSE) and the Determination Coefficient (R^2^). The results show that the prediction accuracy of the TCN-GRU multi-feature fusion model is higher than that of the single-feature models in terms of stability and anti-interference under both operating conditions. The accuracy of the TCN-GRU (three-feature) model is the most optimal in a steady-state condition at 80% of the training set ratio (RMSE = 3.27 × 10^−3^, R^2^ = 0.965). Furthermore, with the increase in the input feature parameter, the TCN-GRU model is closer to the real value, which proves once again that the proposed model can meet the accuracy requirements of the life prediction of the PEMFC.

## 1. Introduction

In recent years, with the aggravation of environmental problems and the energy crisis, more and more countries have realized the importance of clean energy in future energy development, and have started to research and develop infrastructures and energy storage devices around hydrogen energy [1,2,3]. The Proton Exchange Membrane Fuel Cell (PEMFC), a mainstream hydrogen energy power device, is a typical hydrogen fuel cell with high power density, fast startup, long sustainable operation time, and zero pollution and emission [4,5]. Therefore, it has the prospect of wide application in the power field of automobiles, power locomotives, and ships [6].

However, the cost and life of PEMFCs are two important factors limiting their large-scale commercial application [7], and an accurate assessment of durability is key to improving reliability and extending lifespan. Therefore, many scholars have devoted themselves to exploring the life prediction methods for PEMFCs. Nowadays, there are three main methods: model-based method, data-driven method, and hybrid prediction method [8].

The model-based prediction method mainly focuses on modeling from the fuel cell degradation mechanism, which requires less data and has high accuracy. Ou et al. [9] proposed a PEMFC prediction method based on a semi-empirical model to realize the prediction of PEMFC degradation and the estimation of its remaining service life under automotive environmental conditions by introducing electrochemical surface area and equivalent resistance degradation models, respectively. Lechartier et al. [10] presented a combined static and dynamic prediction model and verified the accuracy of the model with experimental data. Ao et al. [11] used a life prediction method based on a frequency-domain Kalman filter (FDKF) and a voltage degradation model. By processing the data in groups, the computation time can be greatly reduced with high accuracy. However, the model-based method requires an in-depth understanding of the aging mechanism of the stack and strong modeling ability. Meanwhile, the internal structure and materials of different stacks are not the same, and the aging mechanism is somewhat different, so some key parameters in the modeling cannot be defined directly, which makes it difficult to establish a complete and accurate mechanism model.

The data-based prediction methods are mainly used to build a system behavior model on PEMFC historical operation data directly for fault diagnosis and life prediction. These methods do not require in-depth analysis of the internal reaction mechanism of the fuel cell, but rather comprehensive analysis of a large amount of historical operation data using a variety of methods, such as statistical modeling, machine learning, deep learning, and hybrid learning [12]. Wu et al. [13] improved on the Relevance Vector Machine (RVM) and introduced the Support Vector Machine (SVM) algorithm for comparison, and the results proved that the improved RVM statistical model can form an adaptation to the prediction process due to the absence of limitations in the kernel function, and the prediction performance is better. Liu et al. [14] compared several life prediction methods based on different structural neural networks, and the results showed that the Adaptive Neuro-Fuzzy Inference System-Fuzzy C-Means (ANFIS-FCM) has the best short-term prediction performance, while the introduction of the Particle Swarm Optimization (PSO) algorithm realizes the automatic adjustment of parameters of ANFIS-FCM. Mezzi et al. [15] designed an echo state network-based variable load prediction method for fuel cells that can be predicted without prior knowledge of the variable load profile. Zuo et al. [16] combined the attention mechanism and GRU for PEMFC prediction, and the results showed that the model has high prediction accuracy on both dynamic and pseudo-stable datasets. However, the data-based prediction method still has the problem of “black box” for the degradation mechanism and state changes of the PEMFC, and there are some limitations in relying on the prediction results to decide the follow-up maintenance measures.

The hybrid prediction methods combine different life prediction methods to improve prediction accuracy by improving individual prediction method weaknesses. There are mainly model–data hybrid-driven methods and data–data hybrid-driven methods [17]. The model–data hybrid-driven method retains the interpretability of the parameters in the model-based method, but also makes full use of the advantages of the two models by blurring them with the help of the data-based method when the mechanism process is not clear. Pan et al. [18] combined model-based AEKF and data-driven NARX with external inputs for predicting PEMFC performance degradation and validated the predictive power of the method using two different datasets. Liu et al. [19] proposed a 2-Stage hybrid prediction method: Stage 1 used an automated machine learning algorithm based on an evolutionary algorithm and an adaptive neuro-fuzzy inference system to achieve voltage degradation prediction in a long time series state. Stage 2 utilized a semi-empirical degradation model based on the predicted data to estimate the remaining lifetime of the battery. The data–data hybrid-driven method can fully utilize the different dimensional information of the algorithm for prediction, thereby improving the prediction accuracy and robustness. Zhu et al. [20] combined Bayesian theory and the self-attention mechanism to propose a B-GRU hybrid model, which can quantify the uncertainty parameters in the prediction process, and experimentally proved that the hybrid algorithm’s computational accuracy is higher than that of the commonly used neural networks when the training data are less than 380 h.

The degradation of the PEMFC’s internal structure is often not accurately characterized by a single feature with poor robustness; the single feature may change due to the performance degradation of the stack, which in turn affects the output characteristics of the stack. And due to the strong coupling of the internal parameters of the PEMFC, it is often difficult to determine the strong correlation parameters using multiple features to predict the degradation trend, which leads to a long prediction time and low prediction accuracy. The Temporal Convolutional Network-Gated Recurrent Unit (TCN-GRU) is an algorithm that combines the ability of the TCN to extract features of time series data with the advantage of GRU for higher computational efficiency in long time series data prediction, which is able to better handle multi-feature data inputs [21], and has been demonstrated in electrical load [22] and lithium-ion battery life prediction [23]. Therefore, this paper proposes a multi-feature fusion method for the life prediction of automotive PEMFCs based on the TCN-GRU. This method is also the first application of the TCN-GRU algorithm to the life prediction of the PEMFC. The correlation of feature parameters in PEMFC experiments is analyzed, and the strength of the correlation between other feature parameters and voltage is projected, so that multiple strongly correlated features are filtered to participate in the fusion life prediction. The TCN-GRU dual-feature and three-feature fusion life prediction models are constructed, and the voltage trend prediction is carried out by utilizing the FCLAB test open dataset and the dynamic cycling operating condition test dataset of the automotive PEMFC system, comparing it with the evaluation indicators to judge the applicability of the models.

The rest of paper is structured as follows. In Section 2, for the characteristics of steady-state operating conditions and dynamic cycling conditions, correlation analysis is performed on the original data, and several strongly correlated feature parameters are filtered and smoothed for noise reduction and reconstruction. Section 3 presents the single-feature parameter and multi-feature parameters fusion prediction models. The life prediction under different operating conditions is carried out in Section 4, and the model evaluation results are analyzed and summarized in Section 5. The conclusions and prospects for future work are summarized in Section 6.

## 2. Multi-Feature Correlation Analysis and Data Processing

### 2.1. Source of Data

**Figure 1:**
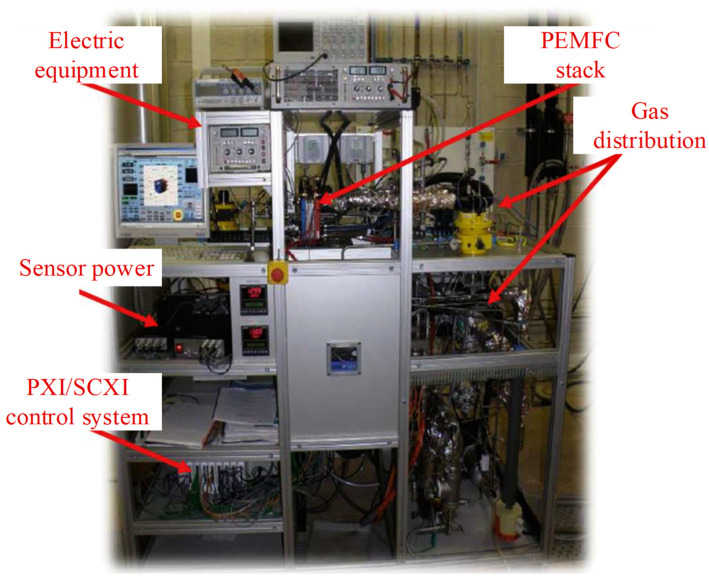
2014 IEEE PHM Data Challenge durability test bench [25,26].

The aging process of internal components of the PEMFC such as the proton exchange membrane and bipolar plate is relatively slow. Meanwhile, due to the continuity of the durability experiments and the closure of the power stack, it is not possible to find a reasonable performance index from the internal perspective, so the external index is used as a symbol for characterization [24]. In order to accurately predict the aging trend of the PEMFC in steady-state conditions, the first PEMFC dataset group (FC1) from the 2014 IEEE Prognostics and Health Management (PHM) Data Challenge durability test public dataset is used [25]. The fuel cell test bench used for this data is shown in Figure 1. The specific parameters of this stack are shown in Table 1. The monitoring parameters include air/hydrogen flow and pressure, coolant temperature, output current, output voltage, monomer voltage, and so on.

**Figure 2:**
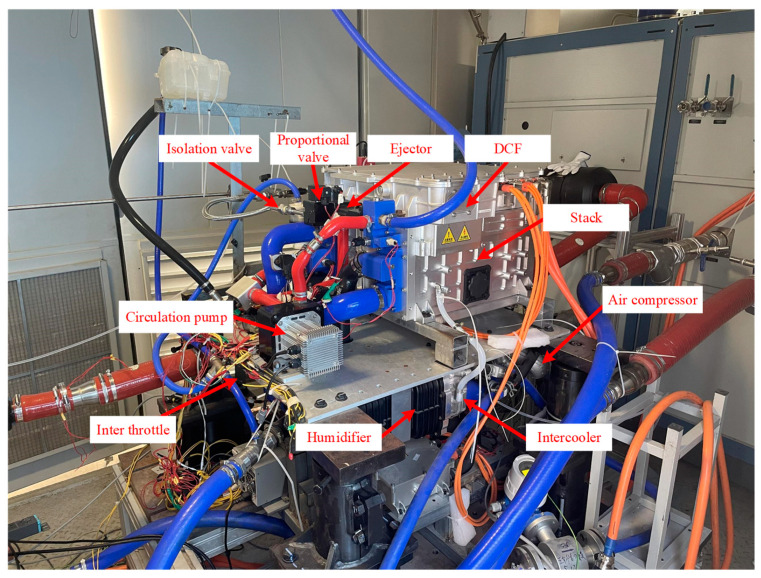
60 kW fuel cell test bench [27].

**Figure 3:**
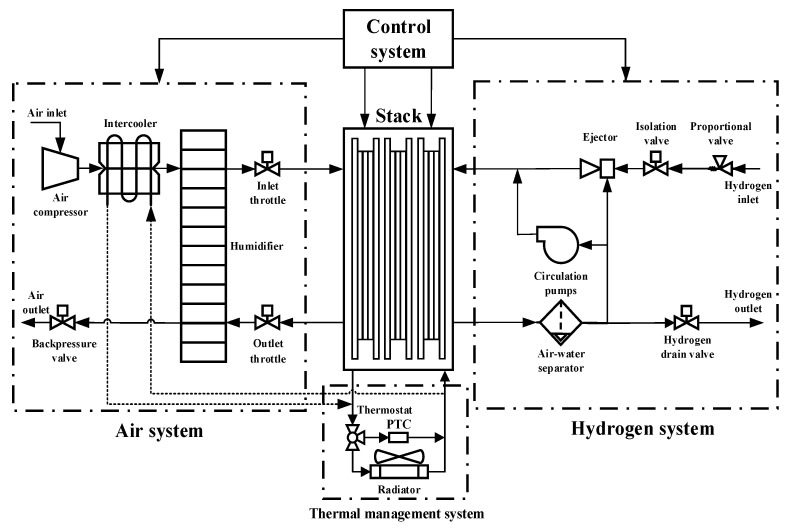
Schematic of the fuel cell system.

The dynamic operating conditions data come from the cycling conditions simulation experiment completed on the 60 kW fuel cell bench test platform [27]. The dynamic cycling test conditions are based on the *China light*-*duty vehicle test cycle*-*passenger car* (CLTC-P) driving conditions [28], with a single cycle duration of 1800 s, divided into three speed intervals: low, medium, and high. The three speed ranges are 10–50 km/h (low range), 50–80 km/h (medium range), and 80–114 km/h (high range). The test rig cumulatively tested and sampled data for 96 complete cycles with a data sampling interval of 2 s. The test bench is shown in Figure 2. The schematic of this fuel cell system is shown in Figure 3, which mainly consists of the stack, the air system, the hydrogen system, the thermal management system, and the control system. The specific parameters of this stack are shown in Table 2. In this experiment, the anode of the stack was supplied with hydrogen in a cyclic mode, with the hydrogen pressure maintained at about 15 bar and the pressure controlled by a proportional valve to control the flow area. The unreacted hydrogen is returned to the anode inlet via a hydrogen circulation pump and an ejector. The cathode air is pressurized, cooled, and humidified, and then flows into the cathode flow channel. The cathode inlet pressure and flow rate can be adjusted by the air compressor, backpressure valve, and exhaust throttle. The parameters that can be directly measured on the test platform include: output voltage, output power, cathode/anode inlet/exhaust pressures, air compressor speed, coolant inlet/outlet temperatures, proportional valve and backpressure valve openings, hydrogen circulating pump speed, and so on.

### 2.2. Correlation Coefficient Analysis Method

Output voltage can be a key feature to characterize the degradation of the PEMFC [29]. Therefore, when selecting other features for joint prediction, it is necessary to filter out the feature parameters that are closely related to the output voltage. There are three main methods for performing correlation analysis: the Pearson correlation coefficient, the Spearman correlation coefficient, and the Kendall correlation coefficient [30].

The Pearson correlation coefficient is applicable to the comparison between two continuous variables and measures the strength and direction of their linear relationship, with a value between −1 and 1. The Pearson correlation coefficient of variables *X* and *Y* is denoted as $\rho_{X,Y}$, as shown in Equation (1) [31]:

(1) $$\rho_{X,Y}=\frac{E((X-\mu_{X})(Y-\mu_{Y}))}{\sigma_{X}\sigma_{Y}}=\frac{E(XY)-E(X)E(Y)}{\sqrt{E(X^{2})-E^{2}(X)}\sqrt{E(Y^{2})-E^{2}(Y)}}$$

here, *µ* is the expected value and *σ* is the standard deviation.

When $\rho_{X,Y}$ = 1, it indicates that there is a completely positive linear relationship between variables *X* and *Y*, i.e., the two variables are completely positively correlated. When $\rho_{X,Y}$ = −1, it means that there is a completely negative linear relationship between variables *X* and *Y*, i.e., the two variables are completely negatively correlated. When $\rho_{X,Y}$ = 0, it means that there is no linear relationship between variables *X* and *Y*, i.e., the two variables are uncorrelated.

The Spearman correlation coefficient is used to determine the monotonic relationship between two variables, which does not require the data to follow a normal distribution, and it calculates the correlation by ranking the variables, which is applicable in the case of a nonlinear relationship. The rank of a number in a variable is the position of that number in the set of columns from smallest to largest. This method is particularly suitable for rank order data. The Spearman correlation coefficient of variables *X* and *Y* is denoted as ρ, which is calculated as shown in Equation (2) [32]:

(2) $$\rho =1-\frac{6\sum_{i=1}^{n}d_{i}^{2}}{n(n^{2}-1)}$$

here, $d_{i}$ is the rank difference obtained by subtracting $Y_{i}$ from $X_{i}$ and n is the number of data points. This method for the degree of linear relationship is consistent with the Pearson correlation coefficient.

The Kendall correlation coefficient is a statistic used to measure the hierarchical relationship between two variables, which does not depend on the specific values of the data, but rather calculates the correlation based on the hierarchy of the data. The Kendall correlation coefficient is particularly applicable when the data do not satisfy a normal distribution or there are outliers. The Kendall correlation coefficient between two variables is denoted as τ, which is calculated as shown in Equation (3) [33]:

(3) $$\tau =\frac{N_{c}-N_{d}}{n\times (n-1)/2}$$

here, $N_{c}$ is the number of two variables that agree in rank, $N_{d}$ is the number of two variables that do not agree in rank, and n is the number of elements.

The feature parameters of the PEMFC are coupled with each other, but do not have a linear relationship with each other, and at the same time, there is no normal distribution law. Therefore, in this paper, the Spearman correlation coefficient and Kendall correlation coefficient are selected as multi-feature filtering methods.

### 2.3. Multi-Feature Filtering and Data Processing

Since the monitoring parameters of the steady-state condition dataset and the dynamic cycling condition dataset are not exactly the same, and the operating environments are different. Therefore, the multi-feature correlation analysis and data processing of the two conditions are performed separately.

#### 2.3.1. Steady-State Condition Multi-Feature Filtering and Data Processing

In the FCLAB FC1 group dataset, the test bench monitors 24 feature parameters. Not all of them affect the degradation of the PEMFC and some of the parameters are repetitive in properties, so the single voltages $U_{1}-U_{5}$, the current density i, and the current I are excluded. Since hydrogen and air are compressible fluids, the relationship between their pressures and flow rates cannot be directly described by Bernoulli’s equation and they cannot be replaced with each other. Therefore, both pressures and flow rates are filtered into the feature parameters to be analyzed. Finally, a total of 17 feature parameters are retained, as shown in Table 3.

**Figure 4:**
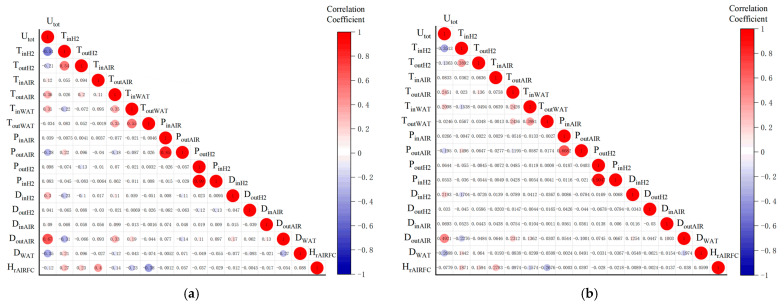
Matrix of correlation coefficients of feature parameters for steady-state condition: (**a**) Comparison of Spearman correlation coefficient; (**b**) Comparison of Kendall correlation coefficient.

The correlation analysis of 17 feature parameters in the FC1 group dataset is shown in Figure 4. When the absolute value of the correlation coefficient is 0.8–1.0, it is called a strong correlation. When the absolute value of the correlation coefficient is 0.3–0.8, it is called weak correlation. When the absolute value of the correlation coefficient is less than 0.3, there is no correlation between the two values [34]. Comparing with the Figure 4a Spearman results, the ρ values of $D_{outAIR}$ and $T_{inH2}$ with $U_{tot}$ are 0.67 and −0.51, respectively, which are higher than the correlation of other feature parameters, and the same results are found in the Figure 4b Kendall correlation coefficient. Therefore, $D_{outAIR}$, $T_{inH2}$, and $U_{tot}$ are selected as the feature parameters for the life prediction of the steady-state operating conditions.

**Figure 5:**
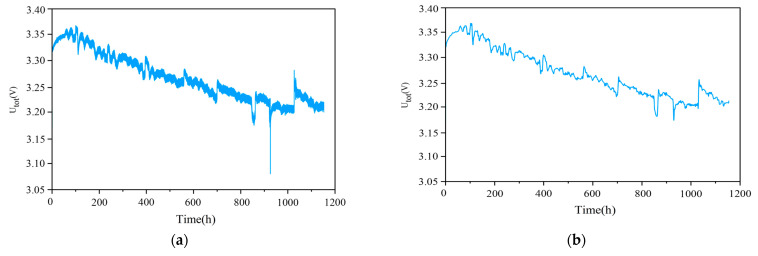

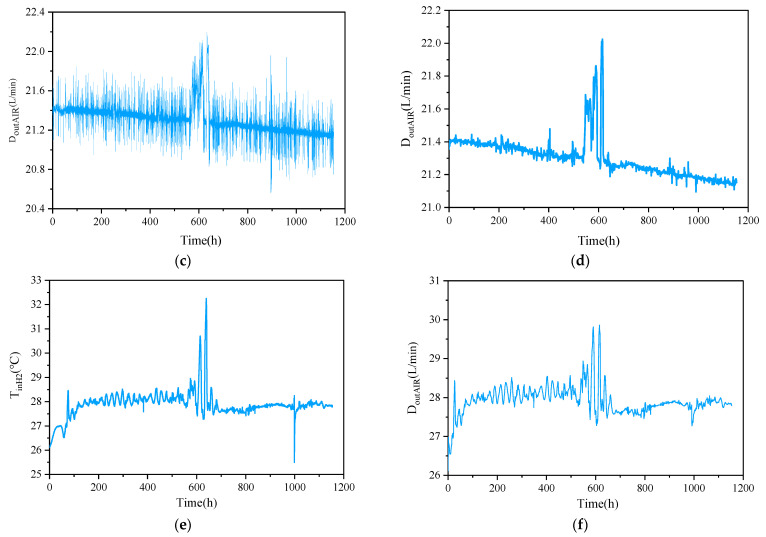
Comparison of DWT smoothing noise reduction results for feature parameters: (**a**) The original $U_{tot}$; (**b**) Results of $U_{tot}$ after smoothing noise reduction; (**c**) The original $D_{outAIR}$; (**d**) Results of $D_{outAIR}$ after smoothing noise reduction; (**e**) The original $T_{inH2}$; (**f**) Results of $T_{inH2}$ after smoothing noise reduction.

If the original FC1 group dataset is used directly for life prediction, it is computationally intensive and highly susceptible to overfitting, so the dataset needs to be reconstructed. Firstly, interval sampling is performed, and the output voltage values are sampled every 70 s. In order to reduce the effect of noise and improve the prediction accuracy, the interval sampling data are smoothed by using a one-dimensional Discrete Wavelet Transform (DWT) [35,36]. The three selected feature parameters before and after the noise reduction and smoothing are shown in Figure 5. As can be seen in Figure 5, the voltage information after the noise reduction process retains the original data change trend, with fewer data anomalies and being overall smoother, which is conducive to model prediction.

#### 2.3.2. Dynamic Cycling Condition Multi-Feature Filtering and Data Processing

The parameters that can be directly monitored in the 60 kW fuel cell bench test platform include output voltage, output power, and more than ten feature parameters. The feature parameters that are not related to the degradation of the PEMFC are also excluded. The final 16 feature parameters retained are shown in Table 4.

**Figure 6:**
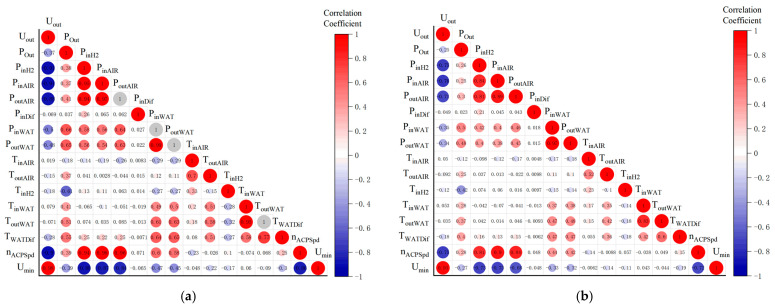
Matrix of correlation coefficients of feature parameters for dynamic cycling condition: (**a**) Comparison of Spearman correlation coefficient; (**b**) Comparison of Kendall correlation coefficient.

Correlation analysis is performed on 16 feature parameters in the dynamic cycling condition dataset, which are shown in Figure 6. In Figure 6a, it can be found that $U_{min}$, $P_{inH2}$, $P_{inAIR}$, $n_{ACPSpd}$, and $P_{outAIR}$ are strongly correlated with $U_{out}$, but the difference is not obvious. From comparing the correlation coefficient in Figure 6b, it can be found that the absolute values of $U_{min}$ and $P_{inAIR}$ are closer to 1, which indicates a strong correlation between these two variables and $U_{out}$. Therefore, $U_{out}$, $U_{min}$, and $P_{inAIR}$ are selected as the features parameters for the life prediction of the dynamic cycling conditions.

**Figure 7:**
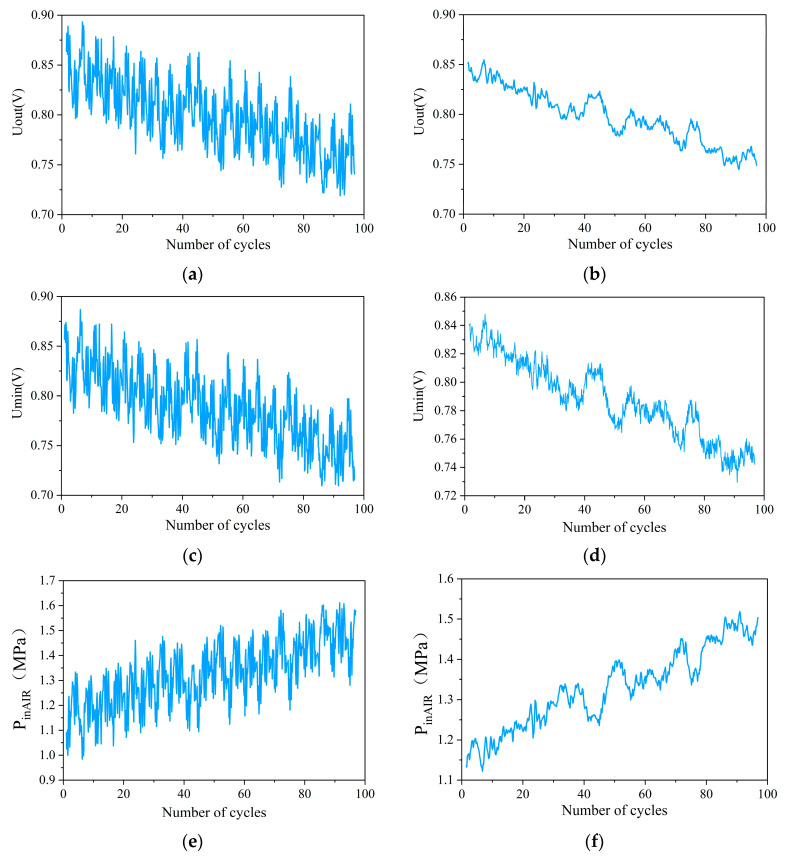
Comparison of reconstruction results of feature parameters based on CGAN: (**a**) The original $U_{out}$; (**b**) Results of $U_{out}$ after reconstruction; (**c**) The original $U_{min}$; (**d**) Results of $U_{min}$ after reconstruction; (**e**) The original $P_{inAIR}$; (**f**) Results of $P_{inAIR}$ after reconstruction.

In the actual operation process, due to the PEMFC being affected by the environment, temperature, operating conditions, its own system, and other factors, the output data collected by the dynamic cycling condition fluctuate, resulting in a large number of outliers [37]. If it is divided by the operating condition trend or data threshold, there may be data overlapping the calculation, which affects the prediction accuracy. Conditional Generative Adversarial Networks (CGANs) is an extension of Generative Adversarial Networks (GANs), which allow the model to introduce conditional information in the process of generating data, so that the generator can generate samples of specific categories or features based on pre-set conditions [38]. Therefore, the original data containing high-frequency noise are reconstructed using the CGAN unsupervised model to make the data preprocessing and feature extraction process adaptive and self-learning. The data before and after processing are shown in Figure 7. It can be found in Figure 7 that all three feature parameters are able to retain the change trend of the original data after data reconstruction, while the noise is significantly suppressed and the curve is smoother.

## 3. Design of Life Prediction Model

The commonly used deep learning algorithms and their usage scenarios are shown in Table 5.

### 3.1. Design of Single-Feature Models

The prediction method with output voltage as a single feature is added to facilitate the comparison and analysis of the advantages and disadvantages of the proposed multi-feature life prediction methods. For the steady-state operating condition life prediction, two prediction methods, the traditional RNN and LSTM, are selected for comparison based on the algorithm characteristics. The RNN model is built based on TensorFlow, and in order to prevent the prediction from overfitting, the Dropout technique is introduced. This technique randomly removes some neurons and their single-step connections during the training process and at each iteration, so that the whole training process is “sparsely” sampled, which avoids the same network from being trained repeatedly and ensures the model’s generalization ability [39]. Meanwhile, in order to ensure that the learning rate η can be adjusted adaptively and maintain the adaptability and stability of each parameter, Adam is chosen as the optimizer, with the number of neurons in the hidden layer being 150. The probability of Dropout random deletion is 0.4, the activation function is ReLU, and the number of iterations is 2000. Considering the training speed and generalization ability, the Batch Size is formulated to be 64 at each iteration.

Based on the LSTM algorithm structure, the Dropout technique is also introduced to prevent predictive overfitting. According to the conclusions of Zhang et al. [40], the Learning Rate η of 0.01 and Dropout random deletion probability of 0.4 are selected to train the network. The number of neurons in the hidden layer of the LSTM is 150, the number of iterations is 2000, and the learning rate is decayed for 50 generations, with a learning rate decay rate of 0.2.

For dynamic cycling operating condition life prediction, a CNN-LSTM-based PEMFC voltage prediction model is selected based on the algorithmic characteristics with the same configuration as above [41,42]. The model utilizes the advantages of the data feature extraction capability of the CNN algorithm and the suitability of the LSTM algorithm for dealing with long-term dependencies. The CNN is mainly used to extract the underlying features of the data to achieve dimensionality reduction, and the use of the LSTM network is mainly to grasp the contextual information more deeply and comprehensively, to avoid the loss of contextual information due to the excessive depth of the CNN network; at the same time, it can allow the model to focus more on the temporal attributes of the data.

### 3.2. Design of Multi-Feature Models

**Figure 8:**
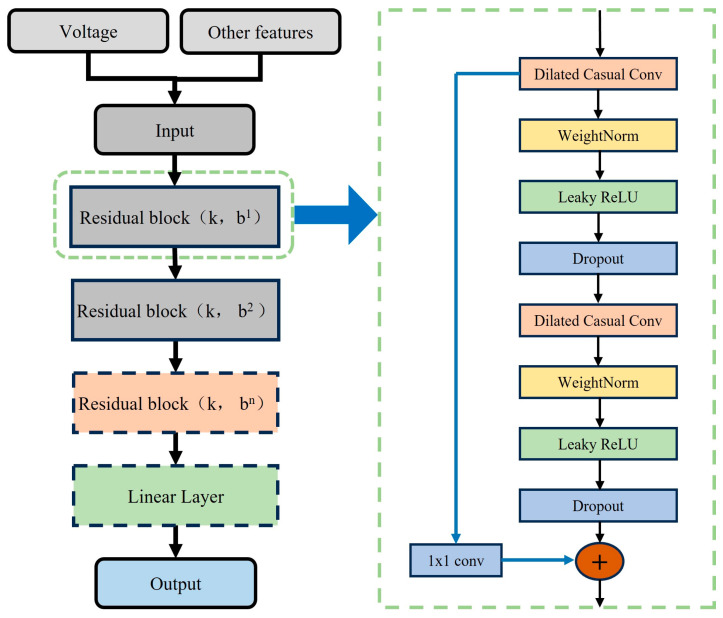
Schematic of the multi-input structure of the TCN model.

Since the essence of multi-feature fusion prediction is the processing of time series features, TCN is selected as the prediction base model in this paper. In order to improve the prediction accuracy of multi-feature data input, the TCN structure is modified, and the improved TCN multi-input model structure is shown in Figure 8. The TCN activation function is replaced with the Leaky ReLU. The Leaky ReLU function introduces a negative slope on the base of ReLU, as shown in Equation (4). Because the Leaky ReLU function has a non-zero gradient, it allows faster convergence during training and avoids the problems of neurons not being updated and gradient vanishing. In order to enhance the model’s capacity and expressiveness [43], three layers of residual blocks are used in the TCN model to help the model capture the long-term correlation relationships within the time series more accurately, while avoiding too many residual blocks leading to model overfitting.

(4) $$f(x)=\left\{\begin{array}{l} x,\;if\;x>0\\ 0.01x,\;otherwise \end{array}\right.$$

**Figure 9:**
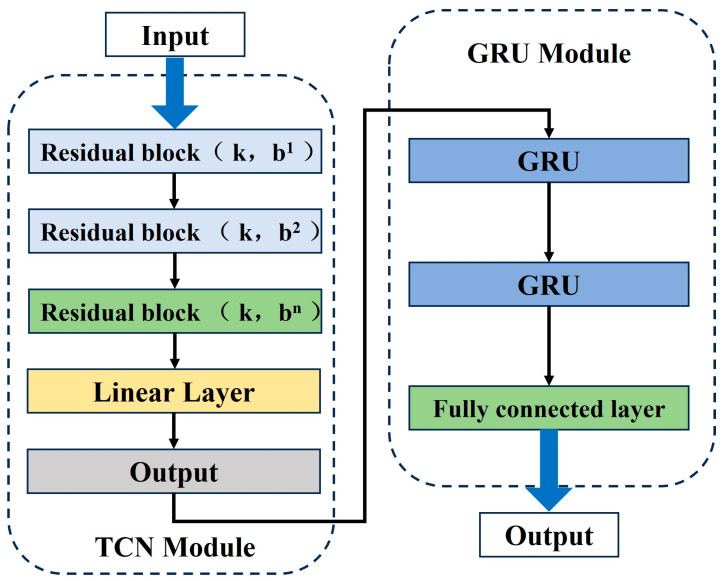
Schematic of the structure of multi-feature fusion life prediction model based on TCN-GRU.

Compared to the LSTM model using three gating mechanisms, the GRU structure is simpler and has fewer hyperparameters. Hence, it has higher computational efficiency in long time series data prediction and is easier to adjust the model hyperparameters and structure [44]. Similarly, in order to better capture multiple sequence features and strengthen the model’s expression ability, two GRU modules are added to the TCN-GRU model. The improved TCN-GRU model is shown in Figure 9. After repeated experimental tuning, the appropriate hyperparameters of the TCN-GRU multi-feature input model are determined, as shown in Table 6.

## 4. Multi-Operating Condition Life Prediction for PEMFCs

In this paper, predictions are made according to the Training Set: Test Set = 4:6 (40%), 6:4 (60%), 8:2 (80%) division. The influence of the experimental environment configuration on the results during the prediction process is minimized as much as possible.

### 4.1. Life Prediction for Steady-State Operating Condition

According to the multi-feature correlation filtering results in the previous section, $U_{tot}$ and $D_{outAIR}$ are selected as the input values for the dual-feature prediction model. $U_{tot}$, $D_{outAIR}$, and $T_{inH2}$ are selected as the input values for the three-feature fusion prediction model.

**Figure 10:**
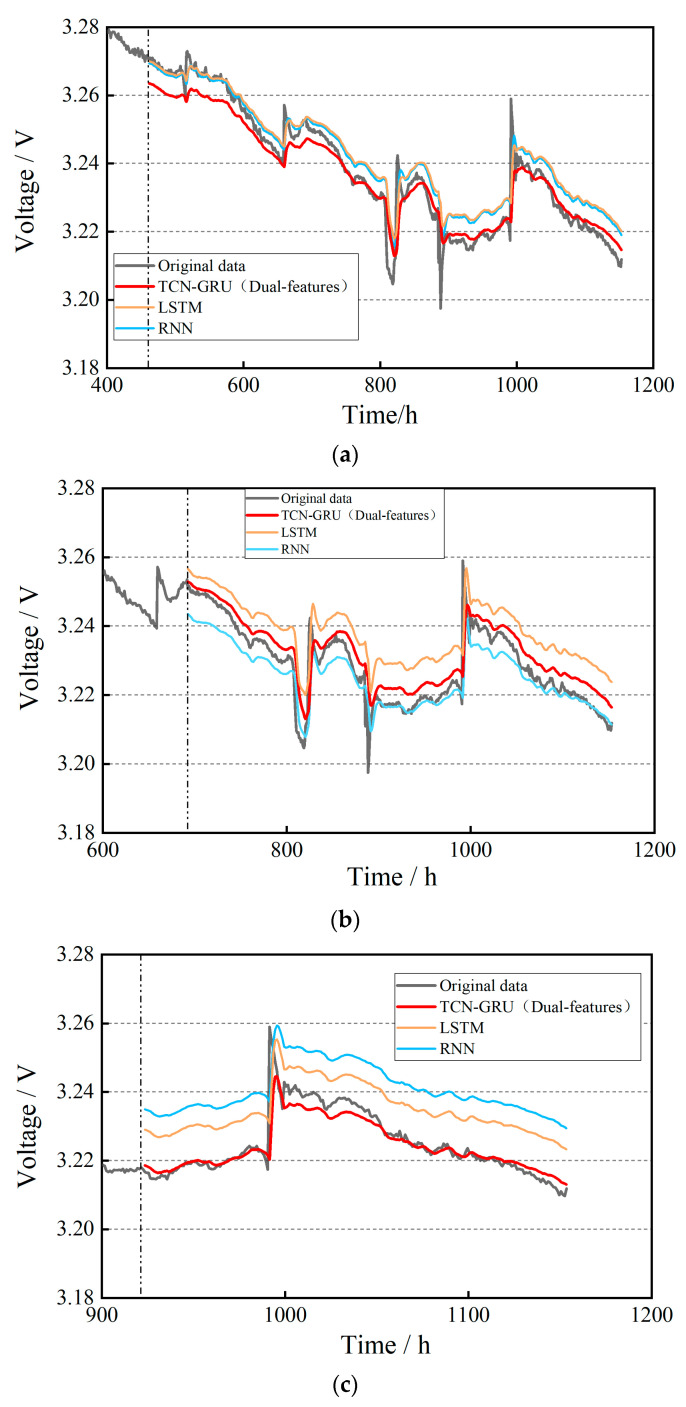
Results of dual-feature fusion life prediction for steady-state operating condition: (**a**) 40% of the training set ratio; (**b**) 60% of the training set ratio; (**c**) 80% of the training set ratio.

The datasets of $U_{tot}$ and $D_{outAIR}$ are imported into the input layer of the TCN-GRU model, and the prediction results under the conditions of 40%, 60%, and 80% of the training set ratio are obtained, respectively, as shown in Figure 10. By analyzing the prediction curves in the figures, it can be found that the results of the TCN-GRU dual-feature fusion prediction model are significantly better than those of the LSTM and RNN models, and the dual-feature fusion prediction fitting performance is better as the number of training sets increases.

**Figure 11:**
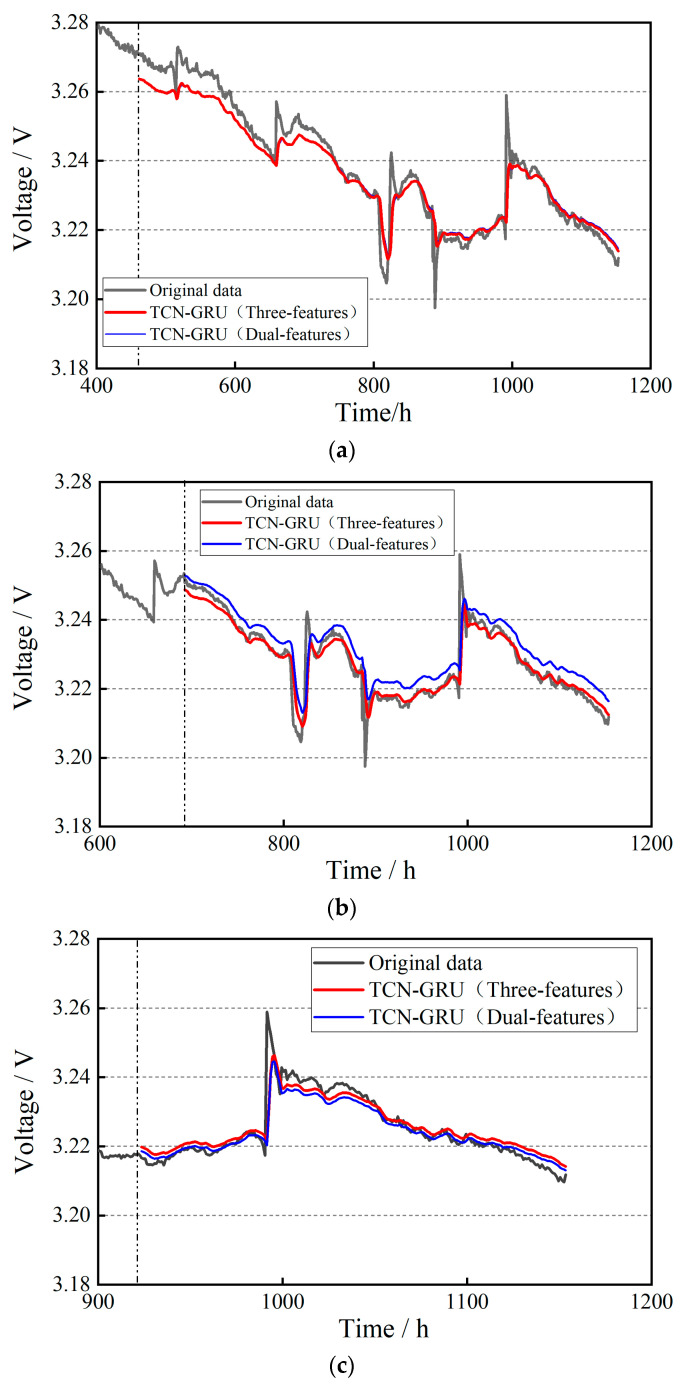
Results of three-feature fusion life prediction for steady-state operating condition: (**a**) 40% of the training set ratio; (**b**) 60% of the training set ratio; (**c**) 80% of the training set ratio.

The datasets of $U_{tot},\;{\;D}_{outAIR}$, and $T_{inH2}$ are imported into the input layer of the TCN-GRU model, and the prediction results are obtained under the conditions of 40%, 60%, and 80% of the training set ratio, respectively, as shown in Figure 11. Analyzing the prediction curves in the figure, it can be observed that the three-feature fusion prediction results are close to the original data at 40%, 60%, and 80% of the training set ratio, indicating that the TCN-GRU model can characterize the degradation inside the stacks more rapidly and accurately and make a prediction after more stacks’ feature parameters are introduced.

### 4.2. Life Prediction for Dynamic Cycling Condition

According to the multi-feature correlation filtering results in the previous section, $U_{out}$ and $U_{min}$ are selected as the input values for the dual-feature prediction model. $U_{out}$, $U_{min}$, and $P_{inAIR}$ are selected as the input values for the three-feature fusion prediction model.

**Figure 12:**
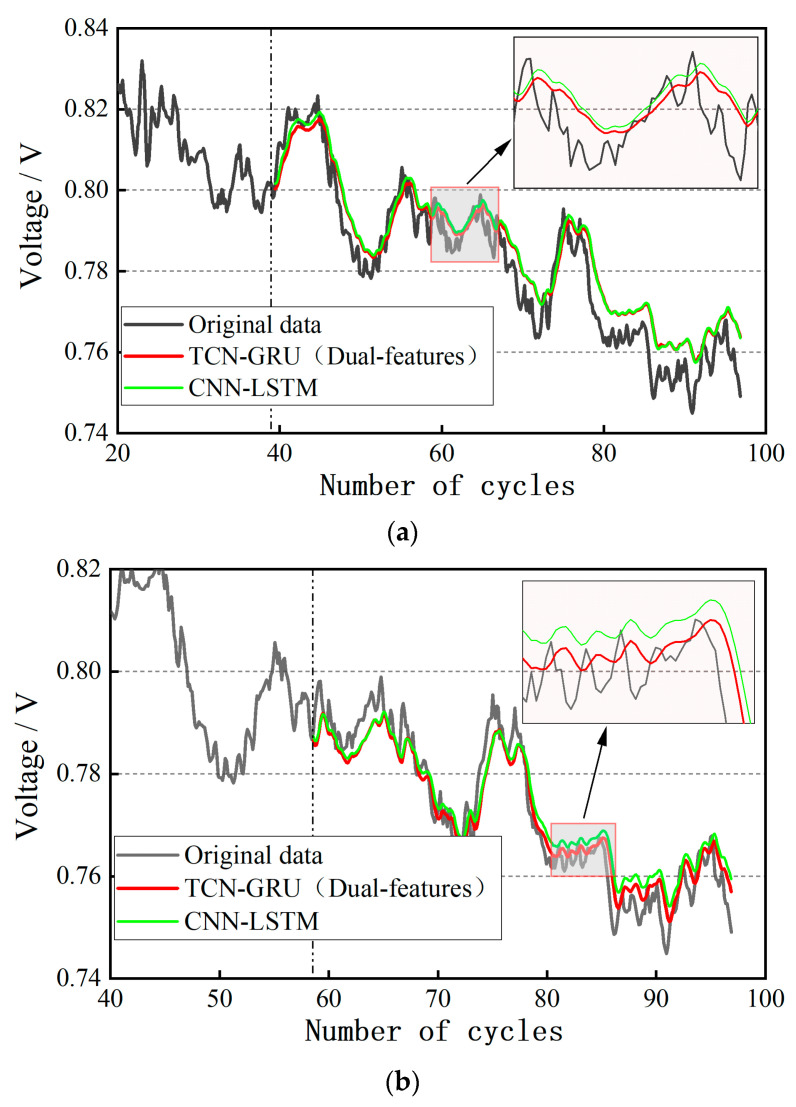

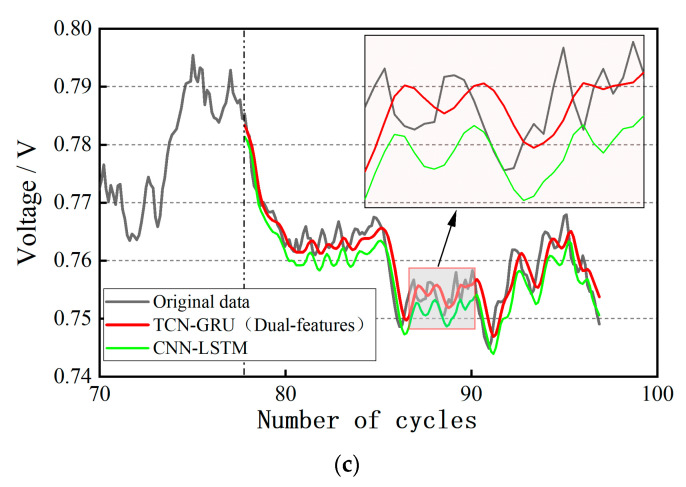
Results of dual-feature fusion life prediction for dynamic cycling condition: (**a**) 40% of the training set ratio; (**b**) 60% of the training set ratio; (**c**) 80% of the training set ratio.

The datasets of $U_{out}$ and $U_{min}$ are imported into the input layer of the TCN-GRU model, and the prediction results under the conditions of 40%, 60%, and 80% of the training set ratio are obtained, respectively, as shown in Figure 12. The dynamic cycling condition of the automotive PEMFC has more uncertainties compared with the steady-state operating condition. Theoretically, the multi-feature fusion prediction has better robustness by combining the trends of multi-feature parameters to comprehensively analyze the degradation characteristics of the PEMFC, and weakening the influence of single-feature parameter anomalies and noise. By qualitatively analyzing Figure 12, it can be seen that the dual-feature fusion prediction is more desirable for dynamic life prediction. From the local zoom in the figure, it can be noticed that the dual-feature prediction curve can better reflect the trend of the voltage value compared with the CNN-LSTM.

**Figure 13:**
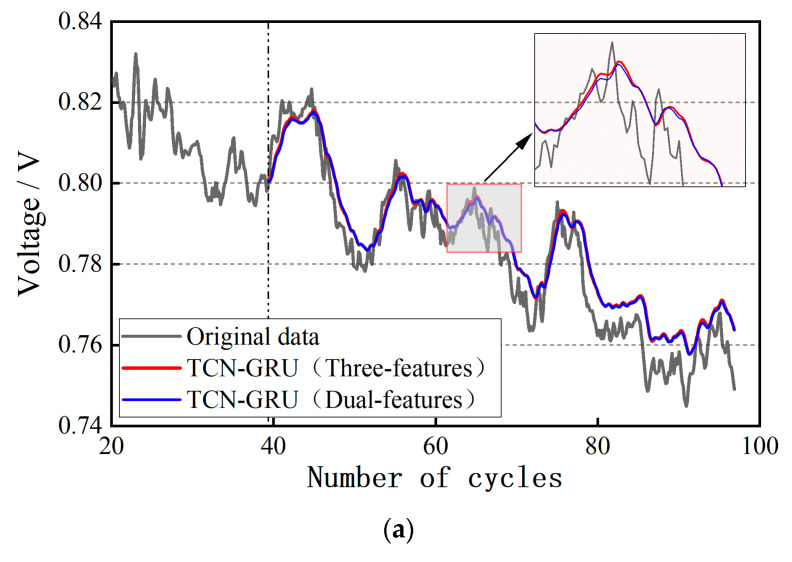

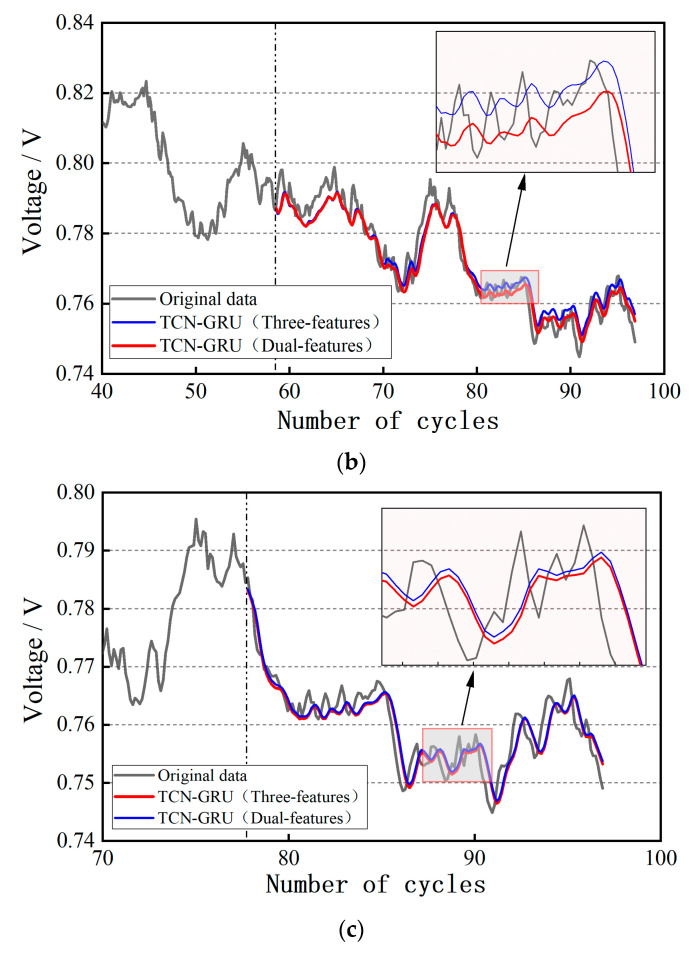
Results of three-feature fusion life prediction for dynamic cycling condition: (**a**) 40% of the training set ratio; (**b**) 60% of the training set ratio; (**c**) 80% of the training set ratio.

The datasets of $U_{out},\;U_{min}$, and $P_{inAIR}$ are imported into the input layer of the TCN-GRU model, and the prediction results are obtained under the conditions of 40%, 60%, and 80% of the training set ratio, respectively, as shown in Figure 13. As can be seen from the figure, the triple-feature fusion prediction is slightly better than the dual-feature fusion prediction, especially when the training set ratio is at 60%. From the local zoom in the figure, the three-feature fusion prediction is closer to the dual-feature fusion prediction, and the fitting effect is better. As the training data volume is larger, the prediction trend for the remaining lifetime is more accurate. At 80% of the training set ratio, the prediction results are almost the same as the original data.

## 5. Results and Discussion

In this paper, predictions are made according to the Training Set: Test Set = 4:6 (40%), 6:4 (60%), 8:2 (80%) division. The influence of the experimental environment configuration on the results during the prediction process is minimized as much as possible. In order to quantitatively assess model performance to visualize the degree of model fitting and prediction ability, it is necessary to use appropriate model evaluation indicators to select, adjust, interpret, and improve the model [45]. According to the characteristics of operating conditions, this paper adopts Root Mean Square Error (RMSE) and the Determination Coefficient (R^2^) as the evaluation indexes of model prediction results.

### 5.1. Analysis of Life Prediction Results for Steady-State Operating Condition

**Figure 14:**
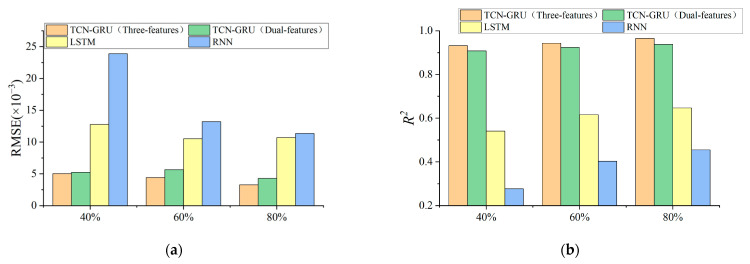
Evaluation of life prediction results for steady-state operating condition: (**a**) Comparison of RMSE results; (**b**) Comparison of R^2^ results.

The prediction evaluation indicators RMSE and R^2^ of the RNN, LSTM, TCN-GRU (dual-feature), and TCN-GRU (three-feature) models are shown in Table 7 and Figure 14. It can be concluded as follows:

- The TCN-GRU (three-feature) has the highest prediction accuracy, with an RMSE value of 3.27 × 10^−3^ and an R^2^ value of 0.965 at 80% of the training set ratio, almost perfectly fitting the original data, which is significantly better than the other two prediction models. It indicates that the multi-feature fusion prediction model’s anti-overfitting and generalization ability is better than the RNN and LSTM models, and it is more suitable for the prediction of long time series and data with multiple parameters.
- The TCN-GRU (three-feature) prediction results are better than the TCN-GRU (dual-feature), compared with the RMSE value reduced by at least 3.82%, and even reduced by 23.6% in the training set ratio of 80%, which indicates that in the long time series prediction, the more features involved in the fusion of the prediction, the greater the model’s ability to resist the abnormal data points or noise, and the better the stability of the model.
- Comparing the TCN-GRU multi-feature fusion prediction results horizontally, with the increase in the training set, the RMSE value of the TCN-GRU (three-feature) decreases by 12.3% and 26.02% in turn, the R^2^ value improves by 1.18% and 2.33%, and the prediction effect becomes better with the increase in the training set, which proves the multi-feature fusion prediction model can meet the requirements of the PEMFC life prediction.

### 5.2. Analysis of Life Prediction Results for Dynamic Cycling Condition

**Figure 15:**
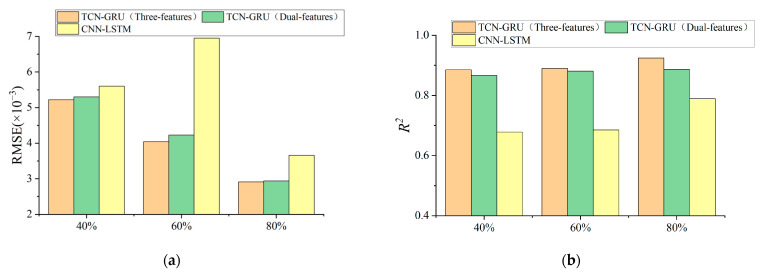
Evaluation of life prediction results for dynamic cycling condition: (**a**) Comparison of RMSE results; (**b**) Comparison of R^2^ results.

The prediction evaluation indicators RMSE and R^2^ of the CNN-LSTM, TCN-GRU (dual-feature), and TCN-GRU (three-feature) models are shown in Table 8 and Figure 15. It can be concluded as follows:

- The TCN-GRU (three-feature) has the highest prediction accuracy, which is slightly better than the TCN-GRU (dual-feature). Comparing the RMSE values, the TCN-GRU (three-feature) model reduces at least 6.79% compared to the CNN-LSTM model, which indicates that the multi-feature fusion prediction model is more resistant to noise and has better robustness in the long time series prediction of dynamic cycling conditions.
- Comparing the R^2^ values, it can be found that the fitting ability of the TCN-GRU multi-feature fusion prediction model has a disconnected improvement compared with the traditional deep learning network. It further shows that a single deep learning network cannot meet the requirements of time series prediction with large data volume, and it is necessary to flexibly combine multiple algorithms, give full play to the advantages of each algorithm, and reasonably build a joint model according to the data volume and data characteristics, in order to achieve better prediction results.

## 6. Conclusions

In this paper, a multi-feature fusion method for life prediction of the PEMFC based on the TCN-GRU is proposed. This model is based on the TCN algorithm that possesses a three-layer residual block, and two GRU modules are added to better capture multiple sequential features and strengthen the model expression capability. To evaluate the model, two commonly used datasets are used for model training under steady-state operating conditions and dynamic cycling conditions, respectively. Strongly correlated feature parameters are imported into the model for prediction after correlation filtering, and RMSE and R^2^ are chosen as the evaluation indicators of the prediction effect. The main conclusions are summarized as follows:

- In the steady-state operating condition, the TCN-GRU (three-feature) has the highest prediction accuracy, with RMSE values of 5.04 × 10^−3^, 4.42 × 10^−3^, and 3.27 × 10^−3^, and R^2^ values of 0.932, 0.943, and 0.965 at 40%, 60%, and 80% of the training set ratio, respectively. Both multi-feature models have better accuracy than the single-feature models. In dynamic cycling conditions, both the TCN-GRU (three-feature) and the TCN-GRU (dual-feature) have better accuracy than the CNN-LSTM. TCN-GRU (three-feature) has the highest prediction accuracy, especially at 60% of training set ratio, with an RMSE value and R^2^ value of 4.04 × 10^−3^ and 0.890, respectively. Compared with the single-feature prediction model, the multi-feature model has many advantages such as strong anti-interference, stability, and high prediction accuracy.
- The results of TCN-GRU multi-feature fusion prediction are compared horizontally. In the steady-state operating condition, the RMSE values of the TCN-GRU (three-feature) decrease by 12.3% and 26.02%, and the R^2^ values improve by 1.18% and 2.33%, in turn, with the increase in the training set. While in dynamic cycling conditions, the RMSE values of the TCN-GRU (three features) reduce by 22.6% and 29.79%, and the R^2^ values improve by 0.6% and 3.8%, respectively. It is fully proved that the proposed multi-feature life prediction method has a better prediction effect as the training set increases, and the prediction model can meet the requirements of PEMFC life prediction.

Therefore, our proposed multi-feature fusion prediction method is suitable for PEMFC lifetime prediction in both steady-state operating conditions and dynamic cycling conditions, with better accuracy than single-feature prediction methods. This method is of significance for improving the accuracy of PEMFC life prediction, extending its service period, and enhancing its reliability and stability. While our study shows that the TCN-GRU outperforms the other three algorithms, since the accuracy of the algorithm depends on the input feature parameters, we have neglected the existence of measurement errors and the influence of other factors on the feature parameters in the actual measurements. Therefore, it is our future research direction to improve the accuracy of the TCN-GRU by reducing the influence of other factors on the accuracy of feature parameters. At the same time, we will continue to try to apply this algorithm to different devices and different complex scenarios, to improve the calculation speed and accuracy, and to realize accurate life prediction and faster and earlier fault diagnosis.

## Figures and Tables

**Table 1:** 2014 IEEE PHM Data Challenge durability test stack parameters [25,26].

Parameters	Value	Unit
Active area	100	cm^2^
Operating temperature	60	°C
Pressure	1.5	bar
Relative humidity	50	%
Load current	70	A
Rated current density	0.7	A/cm^2^

**Table 2:** 60 kW fuel cell test bench stack parameters.

Parameters	Value	Unit
Active area	304	cm^2^
Operating temperature	80	°C
Pressure	2	bar
Relative humidity	80	%

**Table 3:** Feature parameters for steady-state condition.

Feature Parameters	Symbol	Unit
Stack output voltage	$U_{tot}$	V
Inlet temperature of hydrogen	$T_{inH2}$	°C
Outlet temperature of hydrogen	$T_{outH2}$	°C
Inlet temperature of air	$T_{inAIR}$	°C
Outlet temperature of air	$T_{outAIR}$	°C
Inlet temperature of coolant	$T_{inWAT}$	°C
Outlet temperature of coolant	$T_{outWAT}$	°C
Inlet pressure of hydrogen	$P_{inH2}$	Mbar
Outlet pressure of hydrogen	$P_{outH2}$	Mbar
Inlet pressure of air	$P_{inAIR}$	Mbar
Outlet pressure of air	$P_{outAIR}$	Mbar
Inlet flow rate of hydrogen	$D_{inH2}$	L/min
Outlet flow rate of hydrogen	$D_{outH2}$	L/min
Inlet flow rate of air	$D_{inAIR}$	L/min
Outlet flow rate of air	$D_{outAIR}$	L/min
Flow rate of coolant	$D_{WAT}$	L/min
Air humidity	$H_{rAIRFC}$	%

**Table 4:** Feature parameters for dynamic cycling condition.

Feature Parameters	Symbol	Unit
Stack output voltage	$U_{out}$	V
Stack output power	$P_{out}$	W
Inlet temperature of hydrogen	$T_{inH2}$	°C
Inlet temperature of air	$T_{inAIR}$	°C
Outlet temperature of air	$T_{outAIR}$	°C
Inlet temperature of coolant	$T_{inWAT}$	°C
Outlet temperature of coolant	$T_{outWAT}$	°C
Temperature difference of coolant	$T_{WATDif}$	°C
Inlet pressure of hydrogen	$P_{inH2}$	MPa
Inlet pressure of air	$P_{inAIR}$	MPa
Outlet pressure of air	$P_{outAIR}$	MPa
Inlet pressure of coolant	$P_{inWAT}$	MPa
Outlet pressure of coolant	$P_{outWAT}$	MPa
Pressure difference of cathode and anode	$P_{inDif}$	MPa
Speed of air compressor	$n_{ACPSpd}$	rpm
Minimum single voltage	$U_{min}$	V

**Table 5:** Comparison of deep learning algorithms.

Name of Algorithm	Advantage	Drawback	Usage Scenario
Recurrent Neural Network (RNN)	Wide range of applications with internal memory mechanism.	Difficult to parallelize training; prone to gradient vanishing or gradient explosion; computationally inefficient.	Short-to-medium time series
Convolutional Neural Network (CNN)	It is good at capturing local features and can be computed in parallel with strong robustness and generalization ability.	The number of parameters is large, and the convolution operation may lead to information loss.	Spatial data
Long Short-Term Memory Network (LSTM)	Use gating mechanism for easier convergence, stable training, and flexible structure.	The model is large and the computational complexity is high and not easy to interpret.	Memorizing long time series
Temporal Convolutional Network (TCN)	Parallel computation capable; stable gradient propagation; good at capturing long-term dependencies.	Input sequences need to be formulated with a fixed length and are sensitive to sequence ordering.	Long time series
Gated Recurrent Unit (GRU)	The structure is simple, with fewer gating mechanisms, less computational effort, and higher accuracy when the dataset is small.	Weak memory, may not be able to capture long-term dependencies, weak processing of complex sequences.	Simple long time series

**Table 6:** Model hyperparameter settings.

Parameter	Value	Parameter	Value
Neurons	30	Bach size	128
Filters	32	Learning rate	0.001
Sizes	3	Dropout	0.5
Dilations	2	Epoch	30

**Table 7:** Evaluation of life prediction results for steady-state operating condition.

Name of Model	RMSE (×10^−3^)			R^2^		
	40%	60%	80%	40%	60%	80%
RNN	23.91	13.22	11.35	0.277	0.403	0.455
LSTM	12.77	10.51	10.71	0.541	0.616	0.647
TCN-GRU (dual-feature)	5.24	5.67	4.28	0.908	0.924	0.938
TCN-GRU (three-feature)	5.04	4.42	3.27	0.932	0.943	0.965

**Table 8:** Evaluation of life prediction results for dynamic cycling condition.

**Name of Model**	**RMSE (×10^−3^)**			**R^2^**		
	**40%**	**60%**	**80%**	**40%**	**60%**	**80%**
CNN-LSTM	5.60	6.95	3.66	0.678	0.685	0.789
TCN-GRU (dual-feature)	5.30	4.23	2.94	0.866	0.881	0.887
TCN-GRU (three-feature)	5.22	4.04	2.91	0.885	0.890	0.924

## Data Availability

The original contributions presented in the study are included in the article, further inquiries can be directed to the corresponding author.
